# Supplementary material for: Antimicrobial Resistance and Virulence Factors Assessment in Escherichia coli Isolated from Swine in Italy from 2017 to 2021
Source: Pathogens. 2023 Jan 9;12(1):112. doi: 10.3390/pathogens12010112 (PMC9863947; doi:10.3390/pathogens12010112)
Supplement: Supplementary file 1 [file pathogens-12-00112-s001.zip › pathogens-2098110-supplementary.pdf]

pathogens

MDPI

Supplementary Material

Antimicrobial Resistance and Virulence Factors Assessment in *Escherichia coli* Isolated from Swine in Italy from 2017 to 2021

Table S1. Different combination of adhesins and toxins of 457 ETEC isolates, N: number and (%): percentage. STa: heat-stable toxin a; STb: heat-stable toxin b; LT: heat-labile toxin; Stx2e: Shiga toxin 2e.

Adhesins	Toxins																			
	STa		STb		LT		STa, STb		STa, LT		STa, Stx2e		STb, LT		STa, STb, LT		STa, STb, Stx2e		STa, STb, LT, Stx2e	
	N	(%)	N	(%)	N	(%)	N	(%)	N	(%)	N	(%)	N	(%)	N	(%)	N	(%)	N	(%)
**F18**	20	4.4	6	1.3	1	0.2	136	29.8	4	0.9	7	1.5	1	0.2	30	6.6	22	4.8	3	0.7
**F4**	7	1.5	12	2.6	10	2.2	40	8.8	8	1.8	-	-	32	7	35	7.7	-	-	-	-
**F5**	15	3.3	2	0.4	1	0.2	2	0.4	1	0.2	-	-	-	-	1	0.2	-	-	-	-
**F4 F18**	3	0.7	1	0.2	-	-	14	3.1	-	-	-	-	-	-	3	0.7	-	-	-	-
**F41**	8	1.8	6	1.3	-	-	5	1.1	-	-	-	-	-	-	-	-	-	-	-	-
**F5 F41**	14	3.1	-	-	-	-	-	-	-	-	-	-	-	-	-	-	-	-	-	-
**F6**	1	0.2	2	0.4	1	0.2	1	0.2	-	-	-	-	-	-	-	-	-	-	-	-
**F4 F6**	-	-	-	-	-	-	-	-	-	-	-	-	1	0.2	-	-	-	-	-	-
**F5 F6**	-	-	-	-	-	-	1	0.2	-	-	-	-	-	-	-	-	-	-	-	-

Table S2. Percentage (%) of resistance to selected antibiotics from 2017 to 2021 and statistical analysis of observed variations (*r*) listed for all the 826 *E. coli* isolated from swine pathologic samples, and for F18+ and F4+ isolates. Nalidixic acid (NA), amoxicillin and clavulanic acid (AMC), ampicillin (AMP), cefazolin (CZ), enrofloxacin (ENR), florfenicol (FFC); gentamicin (GEN), kanamycin (KAN), tetracycline (TET), trimethoprim + sulfamethoxazole (SXT).

Antibiotics	% of resistant strains					Statistical analysis	
Year of isolation	2017	2018	2019	2020	2021	r	p
NA	84.1	73.6	68.7	69.3	74.2	-0.62	>0.05
AMC	61.7	52.8	63.1	68.9	74.2	0.88	>0.05
AMP	93.5	94.4	95.9	96.1	99.2	0.96	<0.05*
CZ	73.8	83.2	78.3	80.5	79.2	0.37	>0.05
ENR	64.5	64.8	54.4	51	60.8	-0.54	>0.05
FFC	61.7	65.6	63.1	62.3	69.2	0.60	>0.05
GEN	58.9	55.2	64.5	61.9	63.3	0.66	>0.05
KAN	48.6	54.4	63.1	63	59.2	0.76	>0.05
TET	92.5	96	92.6	85.2	85	-0.83	>0.05
SXT	70.1	73.6	78.8	75.1	72.5	0.31	>0.05
N° tested isolates	107	125	217	257	120		
Antibiotics -	% of resistances of F18+ strains					Statistical analysis	
Year of isolation	2017	2018	2019	2020	2021	r	p
NA	90	79.4	68.4	77.6	91.7	0.03	>0.05
AMC	50	38.2	59.2	65.8	79.2	0.87	>0.05
AMP	95	97.1	96.1	93.4	95.8	-0.23	>0.05
CZ	65	91.2	73.7	75	83.3	0.32	>0.05
ENR	55	79.4	42.1	48.7	54.2	-0.36	>0.05
FFC	70	79.4	80.3	81.6	95.8	0.92	<0.05*
GEN	70	79.4	80.3	78.9	91.7	0.88	<0.05*

KAN	50	50	71.1	69.7	91.7	0.94	<0.05*
TET	95	100	96.1	90.8	87.5	-0.79	>0.05
SXT	65	88.2	88.2	85.5	83.3	0.55	>0.05
N° tested isolates	20	34	76	76	24		

Antibiotics -	% of resistances of F4+ strains					Statistical analysis	
Year of isolation	2017	2018	2019	2020	2021	r	p
NA	88	85.2	67.5	73	93.3	-0.02	>0.05
AMC	60	55.6	60	59.5	73.3	0.71	>0.05
AMP	96	92.6	95	94.6	100	0.58	>0.05
CZ	88	81.5	77.5	83.8	66.7	-0.79	>0.05
ENR	72	66.7	57.5	59.5	73.3	-0.10	>0.05
FFC	60	66.7	55	62.2	73.3	0.51	>0.05
GEN	60	51.9	47.5	64.9	66.7	0.50	>0.05
KAN	48	55.6	50	59.5	60	0.81	>0.05
TET	92	96.3	82.5	75.7	86.7	-0.61	>0.05
SXT	68	51.9	60	59.5	73.3	0.35	>0.05
N° tested isolates	25	27	40	37	15		

*the trend was considered statistically significant for p<0.05

Table S3. Percentages (%) of *E. coli* F4+ isolates simultaneously resistant to n° antibiotics (from 1 to 10) and to ≥ 6 antibiotics (multiresistant) from 2017 to 2021. Nalidixic acid (NA), amoxicillin and clavulanic acid (AMC), ampicillin (AMP), cefazolin (CZ), enrofloxacin (ENR), florfenicol (FFC); gentamicin (GEN), kanamycin (KAN), tetracycline (TET) trimethoprim + sulfamethoxazole (SXT). The trend was considered statistically significant for p<0.05.

% of resistances to n° antibiotics of F4+ strains								
		Year of isolation					Statistical analysis	
n° antibiotics	Total	2017	2018	2019	2020	2021	r	p
0	0	0	0	0	0	0	-	-
1	2.1	0	3.7	2.5	2.7	0	-0.09	>0.05
2	2.1	0	0	2.5	5.4	0	0.36	>0.05
3	7.6	4	11.1	7.5	10.8	0	-0.28	>0.05
4	6.3	8	3.7	7.5	5.4	6.7	-0.09	>0.05
5	6.3	12	3.7	7.5	2.7	6.7	-0.50	>0.05
6	13.2	16	14.8	15	8.1	13.3	-0.61	>0.05
7	14.6	8	11.1	20	10.8	26.7	0.75	>0.05
8	16.7	8	14.8	22.5	21.6	6.7	0.09	>0.05
9	14.6	28	22.2	5	10.8	13.3	-0.70	>0.05
10	16.7	16	14.8	10	21.6	26.7	0.69	>0.05
>6	75.7	76	77.8	72.5	73	86.7	0.46	>0.05
N° tested isolates	**144**	**25**	**27**	**40**	**37**	**15**		

Table S4. Virulence factor genes specific primers [22]. Fimbriae (F), heat-labile toxin (LT); heat-stable toxin a (STa); heat-stable toxin b (STb); Shiga toxin (Stx).

Virulence factor	F 5′-3′	R 5′-3′
F4	GTTGGTACAGGTCTTAATGG	GAATCTGTCCGAGAATATCA
F5	AATACTTGTTCAGGGAGAAA	AACTTTGTGGTTAACTTCCT
F6	AAGTTACTGCCAGTCTATGC	GTAACTCCACCGTTTGTATC
F18	TGGTAACGTATCAGCAACTA	ACTTACAGTGCTATTCGACG
F41	AGTATCTGGTTCAGTGATGG	CCACTATAAGAGGTTGAAGC
LTb subunit	GGCGTTACTATCCTCTCTAT	TGGTCTCGGTCAGATATGT
STaP	CAACTGAATCACTTGACTCTT	TTAATAACATCCAGCACAGG
STb	TGCCTATGCATCTACACAAT	CTCCAGCAGTACCATCTCTA
Stx2e	AATAGTATACGGACAGCGAT	TCTGACATTCTGGTTGACTC

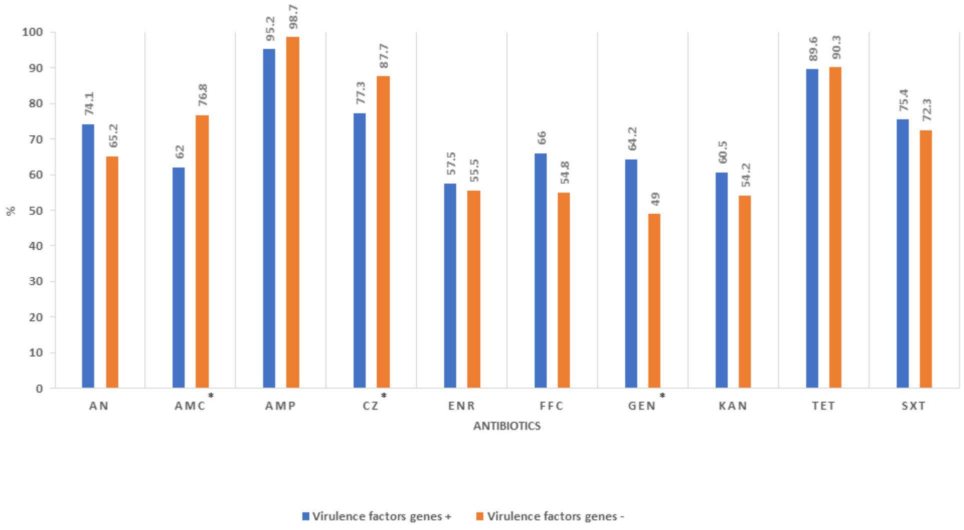

Figure S1. Percentage of resistance to the tested antibiotics of virulence factor genes positive and negative *Escherichia coli*. Nalidixic acid (NA), amoxicillin and clavulanic acid (AMC), ampicillin (AMP), cefazolin (CZ), enrofloxacin (ENR), florfenicol (FFC); gentamicin (GEN), kanamycin (KAN), tetracycline (TET) trimethoprim + sulfamethoxazole (SXT). * Percentage of isolates resistant to antimicrobials with $p<0.01$.

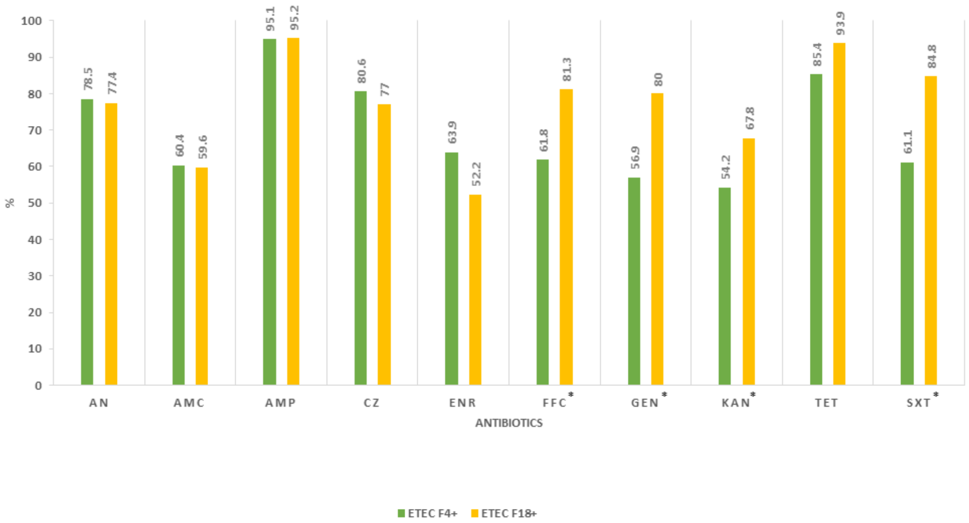

Figure S2. Percentage of resistance to the tested antibiotics of Enterotoxigenic *Escherichia coli* (ETEC) positive to Fimbrial factor (F) 4 or 18. Nalidixic acid (NA), amoxicillin and clavulanic acid (AMC), ampicillin (AMP), cefazolin (CZ), enrofloxacin (ENR), florfenicol (FFC); gentamicin (GEN), kanamycin (KAN), tetracycline (TET) trimethoprim + sulfamethoxazole (SXT). * Percentage of isolates resistant to antimicrobials with $p<0.01$.
